# Validation of Paw Skin Hyperspectral Imaging for Assessing Neuropathic Pain Severity in a Chronic Constriction Injury Model

**DOI:** 10.3390/ijms27125164

**Published:** 2026-06-06

**Authors:** Hsin-Che Wang, Liang-Yi Pan, Jason Sheehan, Meei-Ling Sheu, De-Wei Lai, Ying Ju Chen, Chien-Chia Wang, Hong Lin Su, Hsian-Min Chen, Hung-Chuan Pan

**Affiliations:** 1 Department of Medical Research, Taichung Veterans General Hospital, Taichung 407219, Taiwan; leononwang@gmail.com (H.-C.W.); mlsheu@nchu.edu.tw (M.-L.S.); deweilai123@vghtc.gov.tw (D.-W.L.); hsmin@vghtc.gov.tw (H.-M.C.); 2 Institute of Biomedical Science, National Chung-Hsing University, Taichung 407219, Taiwan; pan0911606850@gmail.com; 3 Department of Neurosurgery, University of Virginia, Charlottesville, VA 22903, USA; jps2f@hscmail.mcc.virginia.edu; 4 Rong Hsing Research Center for Translational Medicine, National Chung-Hsing University, Taichung 407219, Taiwan; suhonglin@gmail.com; 5 PhD Program in Health and Social Welfare for Indigenous Peoples, Providence University, Taichung 407219, Taiwan; yichen5@pu.edu.tw; 6 Department of Life Sciences, National Central University, Taoyuan 407219, Taiwan; superdukewang@gmail.com; 7 Department of Neurosurgery, Neurological Institute, Taichung Veterans General Hospital, Taichung 407219, Taiwan

**Keywords:** hyperspectral imaging, neuropathic pain, chronic constriction injury, CatWalk, thermal hyperalgesia, mechanical allodynia

## Abstract

Neuropathic pain is a debilitating condition lacking objective and quantitative assessment tools, as current evaluations rely largely on subjective reports. Hyperspectral imaging (HSI) is a non-invasive technology that quantifies spatial and spectral tissue characteristics and has been applied in rheumatologic and metabolic disorders. This study investigated whether HSI-detected paw skin alterations correlate with graded nerve injury severity in a chronic constriction injury (CCI) model. Sprague–Dawley rats were assigned to sham or CCI groups with one to four sciatic nerve ligatures. Behavioral assessments (CatWalk XT gait analysis, thermal hyperalgesia, and mechanical allodynia) and paw HSI measurements were performed longitudinally. Histological and molecular analyses were conducted from paw skin to dorsal spinal cord tissues. At 1100 nm, HSI demonstrated progressive and significant spectral deviations proportional to injury severity across all CCI groups, whereas 1300 nm changes were only detected in severe injuries. Histology revealed increased fibrosis, NGF, TNF-α, synaptophysin, and microglial activation with greater injury severity, alongside reduced PGP9.5, neurofilament, AChR, Desmin, GAP-43, Pax3, and BDNF expression. These molecular findings were supported by electrophysiological and behavioral impairments, which correlated with injury grade by HSI. In conclusion, HSI at 1100 nm provides a sensitive and objective indicator of neuropathic pain severity and holds promise as a quantitative translational tool.

## 1. Introduction

Neuropathic pain in humans, resulting from lesions or diseases affecting the somatosensory nervous system, imposes significant and often debilitating effects on health and quality of life [1]. Characterized by persistent pain that is often disproportionate to the original injury, such neuropathic pain is typically resistant to conventional analgesics. Therefore, it contributes to substantial functional impairment and psychological distress [2]. Patients commonly report spontaneous sensation of pain, dysesthesia, and allodynia—symptoms that severely interfere with daily activities such as sleep, mobility, and social interaction [3]. The chronicity of neuropathic pain is strongly associated with elevated rates of anxiety, depression, and cognitive decline, underscoring its profound mental health burden [4]. In terms of financial costs, it places a considerable strain on healthcare systems due to its complex and long-term management, which often require a multidisciplinary approach [5]. Furthermore, it leads to diminished productivity of the workforce, increasing disability, and causing stronger societal impacts [6]. Despite significant advances in understanding its pathophysiology, current treatment strategies remain suboptimal, with many patients experiencing only partial relief—highlighting the urgent need for more effective and targeted interventions [7].

To be more accurate in diagnosis, clinical assessment of neuropathic pain typically involves a combination of patient-reported outcome measures, physical examination, and validated diagnostic tools. Among the most widely recognized instruments are the Douleur Neuropathique 4 (DN4) questionnaire, the Leeds Assessment of Neuropathic Symptoms and Signs (LANSS), and the PainDETECT questionnaire. These tools integrate symptom descriptors, pain characteristics, and, in some cases, physical findings for the differential diagnosis of neuropathic pain [8]. The DN4 scale consists of 10 items divided into two sections. The first section includes seven patient-reported sensory descriptors: burning, painful cold, electric shocks, tingling, pins and needles, numbness, and itching. The second section includes three findings obtained during bedside neurological examination: hypoesthesia to touch, hypoesthesia to pinprick, and pain provoked or increased by brushing. Each positive item is assigned a score of 1, whereas negative responses receive a score of 0, yielding a total possible score ranging from 0 to 10. A total score ≥ 4 is considered indicative of neuropathic pain [8]. The LANSS scale, which incorporates both verbal descriptors and sensory testing (e.g., for allodynia and altered pinprick thresholds), also demonstrates robust sensitivity and specificity [9]. PainDETECT, a self-reported instrument validated in various populations, is especially useful for identifying neuropathic components in chronic low back pain [10]. In specific settings, additional assessments, such as quantitative sensory testing (QST) and intraepidermal nerve fiber density (IENFD) obtained via skin biopsy, are employed to confirm peripheral nerve involvement [11].

However, these tools share inherent limitations. First, while effective for screening, they are not definitive diagnostic methods—particularly when pain presentations are complex or influenced by central sensitization or psychological comorbidities [11]. Second, diagnostic accuracy may be reduced in patients with cognitive impairments, communication barriers, or differences across cultures in interpreting symptom descriptors [12]. Third, physical examinations within these tools (e.g., allodynia testing in LANSS) are susceptible to inter-examiner variability, limiting reproducibility across clinical settings [9]. Fourth, these instruments fail to account for the dynamic nature of neuropathic pain, which may vary over time or present atypically, requiring longitudinal and comprehensive evaluation [11]. As all these tools are subjective—dependent on individual symptom reporting—they should be better interpreted alongside objective clinical findings and, where indicated, supported by confirmatory diagnostic modalities. Therefore, the development of a rapid, objective, and remote assessment instrument for neuropathic pain is imperative.

Peripheral nerve injury often leads to local skin denervation, resulting in increased pain sensitivity and, in some cases, chronic neuropathic pain. Altered innervation during subsequent reinnervation phases may perpetuate hypersensitivity and sensory distortion [13]. Skin biopsies analyzed with immunohistochemistry have revealed changes in specific nerve fiber subtypes—peptidergic (TrkA, CGRP), non-peptidergic (P2X3), and myelinated (NF-200) fibers—allowing for quantification of degeneration and regeneration patterns [14,15,16,17]. These techniques enable the quantification of fiber density across skin layers, revealing differential patterns of degeneration and regeneration post-injury [14,15]. Nonetheless, discrepancies frequently arise between behavioral symptoms, fiber density, and growth factor expression [15], indicating that morphological data alone may not fully explain sensory outcomes. Therefore, developing an in vivo, non-invasive method capable of detecting microstructural or biochemical changes in skin associated with neuropathic pain would represent a major advance.

Hyperspectral imaging (HSI) has recently emerged as a promising optical technology that captures both spatial and spectral data across a wide range of wavelengths. Unlike traditional imaging modalities, HSI can detect signals beyond the visible spectrum, providing detailed insight into tissue composition and microcirculatory physiology [18,19]. When tissue is illuminated with light of varying wavelengths, HSI captures reflected and absorbed spectra that reflect structural and biochemical characteristics of underlying tissue layers [18]. As such, HSI can detect subtle microenvironmental changes in tissue [20]. Recent advancements in imaging analysis and computational power have provided the foundation for applying HSI to medicine. In particular, it has been most extensively studied in the evaluation of burn depth, wound healing progression [21,22,23,24], and skin oxygenation [25]. Its non-invasive nature, high spatial resolution, and capacity to detect subtle physiological alterations make it particularly suitable for studying the skin’s neurovascular and metabolic changes. In the context of neuropathic pain, HSI offers a unique opportunity to non-invasively quantify changes in paw skin reflectance that may correspond to altered vascular perfusion, inflammation, and innervation patterns following nerve injury. Since peripheral nerve lesions often induce cutaneous metabolic and vascular alterations secondary to neurogenic inflammation and sympathetic dysregulation, spectral signatures derived from HSI could serve as objective correlates of neuropathic pain states.

Despite multiple behavioral assays being available for evaluating neuropathic pain in animal models, most of them involve direct animal handling and generate variable results [26,27,28,29,30]. To address the need for remote and reproducible measurement strategies, current research examines the application of HSI to assess skin alterations as a surrogate marker of neuropathic pain. Our aim was, using a chronic constriction injury (CCI) model in animals, to determine the relationship between HSI-derived paw skin signals and behavioral, inflammatory, and electrophysiological changes. We correlated HSI data together with results from the CatWalk gait analysis, degree of inflammation, and nerve conduction alterations across a gradient of sciatic nerve ligations (referring to the number of loose chromic gut ligatures placed around the sciatic nerve to induce graded levels of chronic constriction injury severity). We explored the potential of using HSI as a novel, non-contact tool for neuropathic pain assessment, with translational value for future clinical application.

## 2. Results

### 2.1. A 1100 nm Wavelength in HSI Was the Most Reliable Predictor for Assessing the Severity of Injury

**Figure 1:**
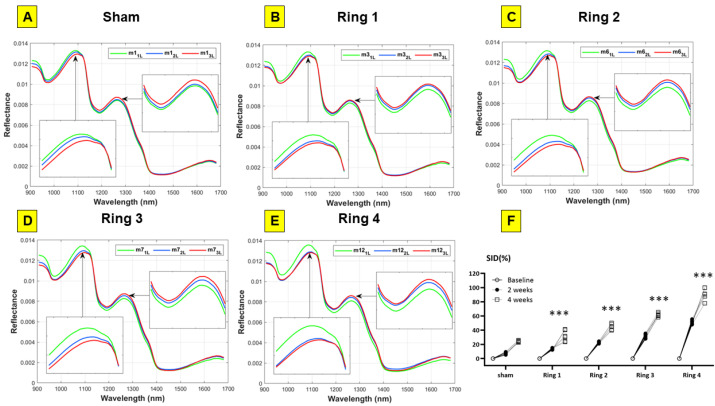
SID changes across varying degrees of nerve compression over time, measured by HSI. Sprague–Dawley rats (250–300 g) were randomly assigned to five groups: sham and one-, two-, three-, or four-ligation groups using 3-0 chromic gut sutures loosely tied around the left sciatic nerve. Hyperspectral imaging (HSI) measurements were performed three days before surgery and then weekly for four weeks post-ligation. (**A**–**E**) Spectral information divergence (SID) values at baseline, 2 weeks, and 4 weeks at various wavelengths in the sham (**A**), one-ligation (**B**), two-ligation (**C**), three-ligation (**D**), and four-ligation (**E**) groups. (**F**) Quantitative comparison of SID values at 1100 nm across all groups over time. *** *p* < 0.001. *n* = 6 per group. See text for SID definition and sham, Ring 1, Ring 2, Ring 3, and Ring 4 groups.

There were a total of five groups of animals, including the sham group and those with one to four rings of CCI. These animals underwent HSI examinations 3 days prior to CCI and again on days 7, 14, 21, and 28 post-CCI. After adjusting the time frame to baseline, two weeks, and four weeks, SID showed a significant difference at the wavelength of 1100 nm in CCI animals with one to four rings, whereas at 1300 nm, a similar significance difference in SID was observed only in CCI animals with three and four rings (Figure 1).

The short-wavelength infrared (SWIR) light (900–1700 nm) penetrates biological tissue to depths of approximately 1–8 mm, depending on the specific wavelength [31]. Consequently, signals detected around 1100 nm predominantly reflect optical alterations within the skin and superficial dermal layers, as well as physiological responses associated with changes in adjacent neural activity after nerve damage. These spectral variations may correspond to alterations in collagen architecture and tissue water content, and other pathophysiological changes induced by nerve damage. Collectively, these findings suggest that HSI at 1100 nm represents a promising quantitative and objective modality for evaluating skin alterations associated with neuropathic pain, with high sensitivity for distinguishing different levels of injury severity.

### 2.2. Paralleled Severity of Histomorphology Alteration in Paw Skin Related to Intensity of Nerve Injury

**Figure 2:**
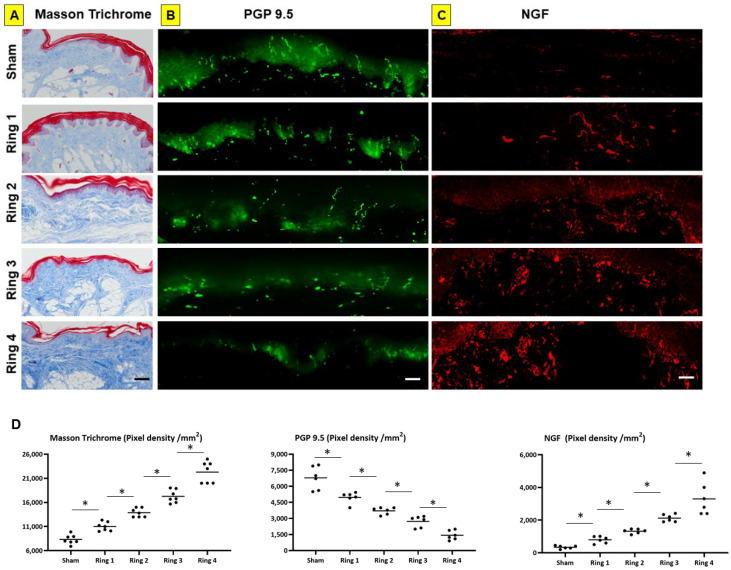
Differential expression of fibrosis, PGP 9.5, and NGF in paw skin 28 days post-injury. Skin samples from the hind paw were harvested on day 28 post-injury. (**A**) Representative Masson’s trichrome staining showing fibrosis in the sham and ligation groups. (**B**) Immunohistochemistry for PGP 9.5 (a neuronal marker) in the same groups. (**C**) Immunohistochemistry for NGF expression across groups. (**D**) Quantitative analysis of immunoreactivity. * *p* < 0.05 between adjacent groups. Scale bar = 200 μm. *n* = 6 per group. See text for sham, Ring 1, Ring 2, Ring 3, and Ring 4 groups.

An increase in Masson’s trichrome staining intensity reflected the progression of tissue fibrosis. Conversely, a reduction in PGP 9.5 expression corresponded to greater nerve degeneration, while enhanced NGF immunoreactivity in epidermal and dermal axons was associated with heightened neuropathic pain severity [28,32]. In the present study, increased blue staining on Masson’s trichrome, representing collagen deposition and fibrotic connective tissue accumulation within the subcutaneous layer, was strongly correlated with the severity of nerve injury. More severe injury, induced by a greater number of sciatic nerve ligations, resulted in a progressively increased density of blue staining (Figure 2A,D; Table 1). In addition, PGP 9.5 immunoreactivity in the intraepidermal and dermal nerve fibers, reflecting the density of nerve fibers, progressively declined with increasing numbers of constriction rings applied to the sciatic nerve. In contrast, nerve growth factor (NGF) expression exhibited an opposite trend, showing progressive upregulation in parallel with injury severity (Figure 2B–D; Table 1). Collectively, these results demonstrate that fibrotic, neural, and inflammatory alterations in paw skin correspond to the severity of nerve injury and are consistent with the changes detected in spectral information divergence (SID) measurements at the 1100 nm wavelength.

### 2.3. Correlation of Severity of Histomorphology Alteration in Nerve, Dorsal Root Ganglion, and Dorsal Spinal Cord with Intensity of Nerve Injury

**Figure 3:**
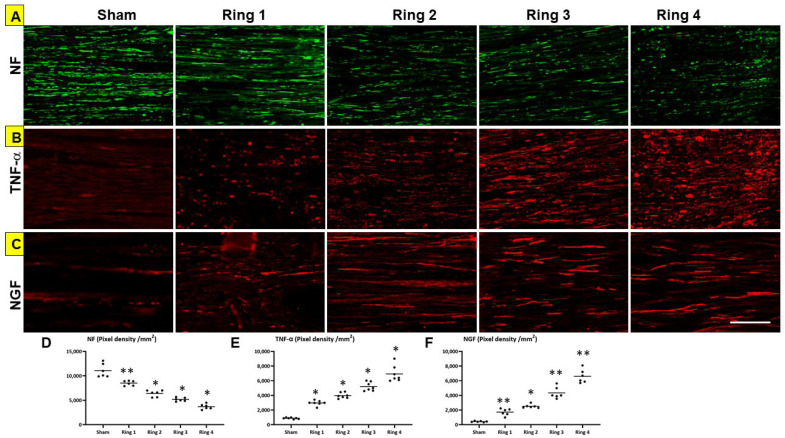
Immunohistochemical analysis of neurofilament, TNF-α, and NGF at the distal sciatic nerve. The sciatic nerve distal to the injury site was harvested on day 28. (**A**–**C**) Representative immunostaining for neurofilament (NF), TNF-α, and NGF across sham and ligation groups. (**D**–**F**) Quantitative analysis of immunoreactivity. * *p* < 0.05; ** *p* < 0.01 between adjacent groups. *n* = 6. Scale bar = 200 μm. See text for sham, Ring 1, Ring 2, Ring 3, and Ring 4 groups.

Neurofilament expression serves as a mature marker for nerve myelination and is inversely related to the expression of inflammatory cytokines such as TNF-α and NGF [28]. In this study, neurofilament expression in the distal segment of the injured nerve, located 1 cm distal to the ligation site, progressively decreased as the number of constriction rings increased. In contrast, the expression of TNF-α and NGF increased in parallel with the severity of nerve constriction injury. The sham group demonstrated the highest density of neurofilament-positive axonal fibers, whereas a progressive reduction was observed with increasing numbers of nerve ligations. Elevated expression of TNF-α and NGF reflected the inflammatory and regenerative responses following nerve injury. Only minimal expression of these markers was observed in the sham group; however, their expression increased progressively in proportion to the severity of nerve constriction injury (Figure 3A–F).

**Figure 4:**
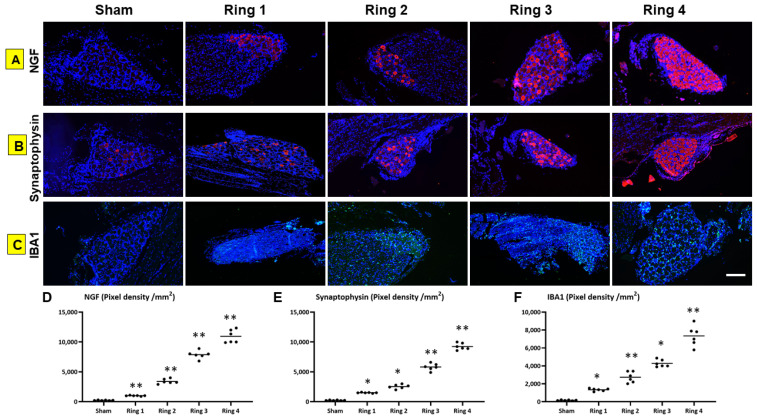
Inflammatory markers in dorsal root ganglia following graded chronic constriction injury. Dorsal root ganglia were harvested 28 days post-surgery. (**A**–**C**) Representative immunofluorescent images of NGF (green), synaptophysin (red), and Iba-1 (red), with DAPI nuclear staining (blue) across groups. (**D**–**F**) Quantitative analysis of immunoreactivity. * *p* < 0.05; ** *p* < 0.01 between adjacent groups. *n* = 6. Scale bar = 200 μm. See text for sham, Ring 1, Ring 2, Ring 3, and Ring 4 groups.

Dorsal root ganglia (DRG) were subsequently harvested and subjected to immunohistochemical staining for NGF, synaptophysin, and Iba-1 to evaluate neuronal and inflammatory responses after nerve injury. In the sham group, only subtle expression of NGF and synaptophysin was detected. In contrast, a stepwise increase in the expression of both markers was observed from the Ring 1 to Ring 4 groups, corresponding to increasing injury severity (Figure 4A,B). Similarly, only minimal accumulation of Iba-1-positive immunoreactive cells was identified in the sham DRG tissues. Nerve constriction injury markedly increased Iba-1 expression in the DRG, with the degree of elevation closely paralleling the severity of injury (Figure 4C). Quantitative analyses further confirmed these histopathological observations (Figure 4D–F).

**Figure 5:**
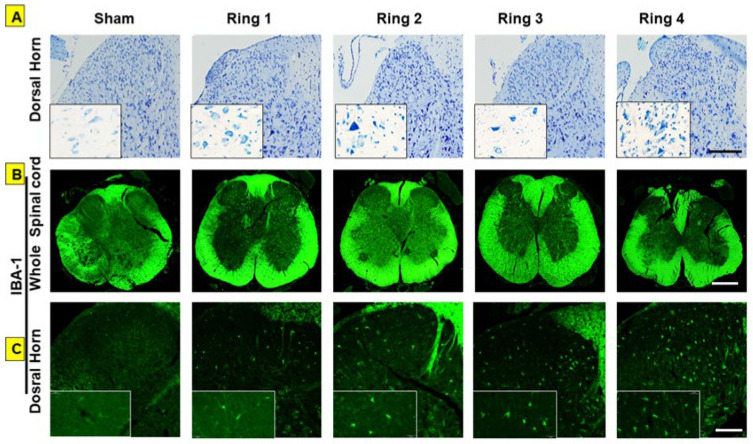

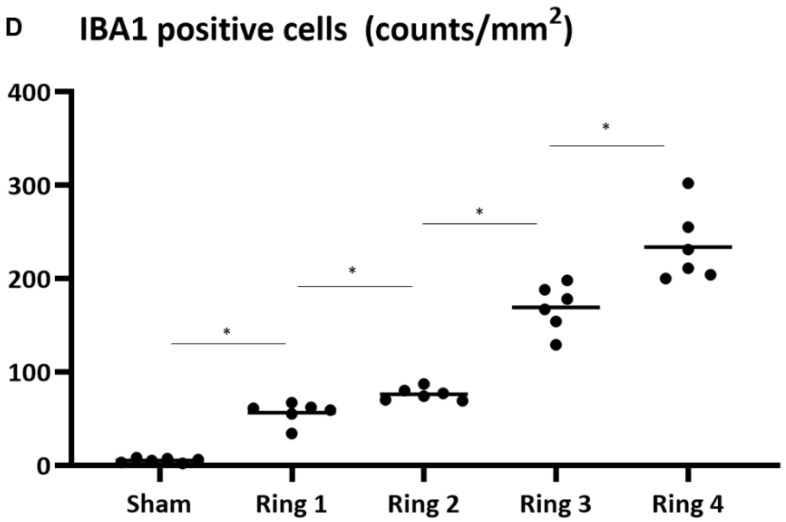
Spinal cord inflammatory responses post-nerve injury. Spinal cord tissue was collected on day 28. (**A**) Representative Nissl staining across groups (scale bar = 200 μm). Cell morphology is seen in the magnification box in the lower left quadrant. (**B**) Iba-1 immunostaining across whole spinal cord sections (scale bar = 500 μm). (**C**) Magnified views of Iba-1 expression in the dorsal horn (scale bar = 200 μm). Cell morphology is seen in the magnification box in the lower left quadrant. (**D**) Quantification of IBA1-positive cells in the dorsal spinal cord. Sham, Ring 1, Ring 2, Ring 3, and Ring 4: see text. * *p* < 0.05. *n* = 6.

Increased aberrant innervation and neuron alteration within the dorsal column were indicative of the severity of nerve damage. Nissl staining is a widely used histochemical technique for studying neuronal morphology and pathology by highlighting rough endoplasmic reticulum (Nissl bodies) in cell bodies. There was subtle staining of Nissl bodies in the sham group. The enhanced cellular clustering in the dorsal spinal cord, as revealed by Nissl staining, displayed a progressive trend related to the degree of nerve injury (Figure 5A).

CCI induces activation of microglia within the dorsal horn of the spinal cord and activated microglia undergo morphological transformation from a ramified resting state to an enlarged amoeboid phenotype. This microglial activation contributes to central sensitization by enhancing excitability of dorsal horn neurons, thereby promoting mechanical allodynia and thermal hyperalgesia associated with neuropathic pain. In the sham group, there was subtle IBA1 cell deposition; elevated Iba1 expression in the dorsal column was significantly associated with the extent of nerve damage (Figure 5B,C). Quantitative analysis of Iba1 expression further substantiated these observations (Table 2).

### 2.4. Correlation Between Anterior Horn Cell Loss and Muscle Damage in Relation to the Severity of Nerve Constriction

**Figure 6:**
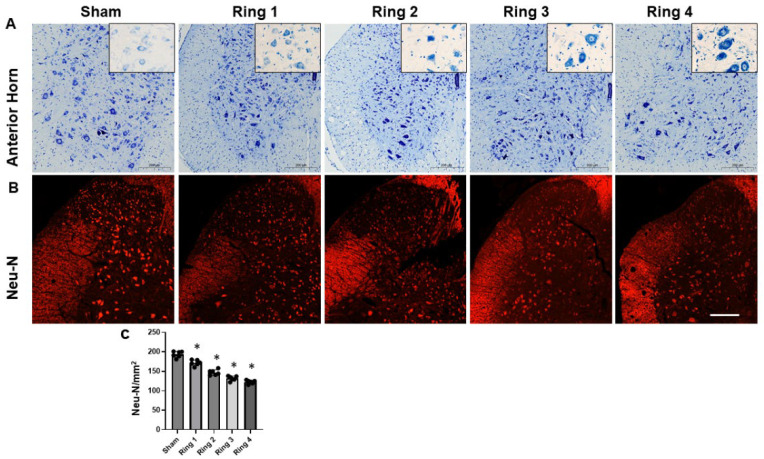
Anterior horn cell loss following sciatic nerve compression. Spinal cord sections were examined at day 28. (**A**) Nissl-stained sections showing anterior horn neuron integrity across groups. Cell morphology is seen in the magnification box in the upper right quadrant. (**B**) NeuN-positive cells indicating neuronal survival. (**C**) Quantification of NeuN-positive neurons. * *p* < 0.05 between adjacent groups. *n* = 6. Scale bar = 200 μm. Sham, Ring 1, Ring 2, Ring 3, and Ring 4: see text.

**Figure 7:**
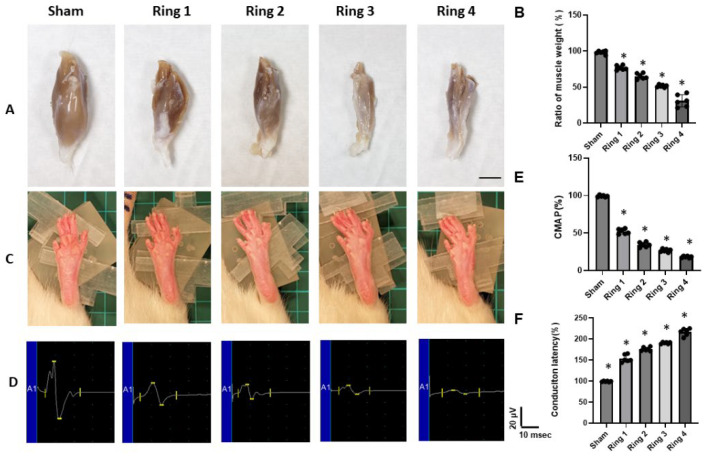
Correlation between muscle atrophy, electrophysiology, and nerve injury severity. (**A**) Harvested muscles from the hindlimbs across groups. (**B**) Quantitative analysis of muscle weight ratio (left/right). (**C**) HSI image of the left paw. (**D**) Electrophysiological recordings of CMAP and nerve conduction velocity. CMAP amplitude was defined as the distance from the peak of the positive waveform to the trough of the negative waveform. Nerve conduction velocity was determined from the onset latency of the positive waveform to the end latency of the negative waveform. The units for voltage and time are indicated in the lower right corner. (**E**,**F**) Quantitative analysis of CMAP amplitude and latency (ipsilateral/contralateral ratio). * *p* < 0.05 between adjacent groups. *n* = 6. Bar = 10 mm for gross photos; 200 μm for histology. Sham, Ring 1, Ring 2, Ring 3, and Ring 4: see text.

We found that nerve damage induced by chronic constriction injury (CCI) not only resulted in muscle denervation, but it also led to the loss of anterior horn cells. Nissl staining revealed nuclear clumping within the anterior horn (Figure 6A), which progressively worsened with the increasing severity of nerve compression (Figure 6B,C). To further evaluate muscle damage and regenerative potential, denervated muscles were harvested for weight measurement and immunohistochemical and electrophysiological analyses. Muscle weights showed an inverse correlation with the number of constrictive rings applied in the CCI model, consistent with morphological changes observed in the paw (Figure 7A–C). Amplitude of compound muscle action potential (CMAP) followed a similar decreasing trend like muscle weight, while nerve conduction latency exhibited an opposite, increasing pattern (Figure 7D–F).

**Figure 8:**
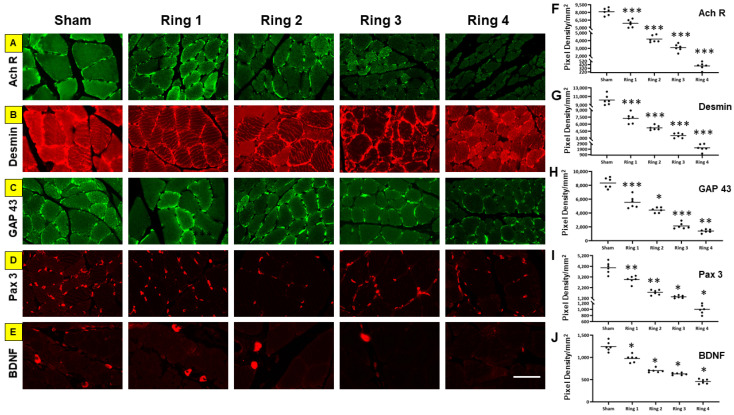
Immunohistochemical evidence of skeletal muscle degeneration following nerve injury. Denervated muscles were collected on day 28. (**A**–**E**) Immunohistochemistry for AChR, Desmin, GAP-43, Pax3, and BDNF in sham and ligated groups. (**F**–**J**) Quantitative analysis across groups. * *p* < 0.05; ** *p* < 0.01; *** *p* < 0.001. Scale bar = 200 μm. *n* = 6. Sham, Ring 1, Ring 2, Ring 3, and Ring 4: see text.

The target markers associated with muscle regeneration included AChR-positive motor end plates, Desmin-positive muscle fibers, GAP-43-positive regenerative structures, Pax3-positive satellite/progenitor cells, and BDNF-positive regenerative muscle components. Immunohistochemical analysis of muscle specimens demonstrated a progressive decline in the expression of AChR, Desmin, GAP-43, Pax3, and BDNF as the severity of nerve constriction increased (Figure 8A–E). Quantitative analysis further confirmed a consistent reduction in the expression of these regenerative markers across the experimental groups (Figure 8F,G).

### 2.5. Representative Neurobehavioral Alterations Associated with Varying Intensities of Nerve Compression

**Figure 9:**
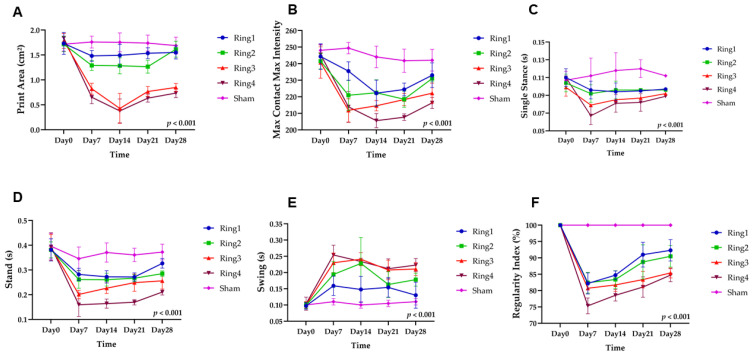
Plots of various parameters of XT CatWalk system alteration related to different severities of nerve compression at different time points. Sprague–Dawley rats (250–300 g) were randomly assigned to five groups: sham, and one, two, three, or four 3-0 chromic gut ligatures loosely tied around the left sciatic nerve. All XT Catwalk gait analyses were measured 3 days before the ligation, and continuous measurements were made once a week for 4 weeks post-surgery until the end of the experiments. (**A**) Plot of the intensity of the printed area related to the different intensities of nerve compression at different time points. (**B**) Plot of the maximum contact maximum intensity related to the different intensities of nerve compression at different time points. (**C**) Plot of the stand phase related to the different intensities of nerve compression at different time points. (**D**) Plot of the swing phase related to the different intensities of nerve compression at different time points. (**E**) Plot of the single stance related to the different intensities of nerve compression at different time points. (**F**) Plot of the regular index related to the different intensities of nerve compression at different time points. Sham, Ring 1, Ring 2, Ring 3, and Ring 4: see text. *n* = 6. Statistical analysis by repeated-measures ANOVA. *n* = 6 per group.

In this study, CCI was induced on one side using different numbers of ligatures to simulate different severities of nerve damage. Neurobehavioral assessment was performed using XT CatWalk gait analysis, with multiple gait parameters evaluated. Print area represented the surface area of paw contact during locomotion and reflected weight-bearing capacity. Stance phase indicated the duration of paw contact with the walkway, whereas swing duration represented the period during which the paw remained off the surface. Maximum contact intensity reflected the peak pressure exerted during paw placement and served as an indicator of limb loading and motor function. The regularity index was used to evaluate interlimb coordination and gait consistency. For each animal, at least three compliant runs with consistent walking speed were selected for analysis, and the average value was calculated. Progressive deterioration in gait coordination and weight-bearing capacity was observed with increasing severity of nerve constriction, as evidenced by reduced print area, decreased maximum contact intensity, shortened stance phase, prolonged swing duration, and lower regularity index values. Statistically significant differences were observed across injury levels in several key parameters, including print area (Figure 9A), maximum contact intensity (Figure 9B), stance phase duration (Figure 9C), swing phase duration (Figure 9D), single stance duration (Figure 9E), and regularity index (Figure 9F).

Mechanical allodynia and thermal hyperalgesia are hallmark indicators of neuropathic pain. These were assessed using the Von Frey filament test and the hot plate assay, which are widely employed to evaluate mechanical and thermal sensitivity in neuropathic pain models. The Von Frey filament test was used to evaluate mechanical allodynia following nerve injury. Briefly, calibrated filaments were applied perpendicularly to the plantar surface of the hind paw until bending occurred, and the paw withdrawal threshold was recorded as an indicator of mechanical sensitivity. The hot plate assay was performed to assess thermal nociceptive responses. Animals were placed on a heated plate maintained at a constant temperature, and the latency to paw withdrawal, licking, or jumping was recorded. A shortened withdrawal latency indicated increased thermal hyperalgesia after nerve injury.

**Figure 10:**
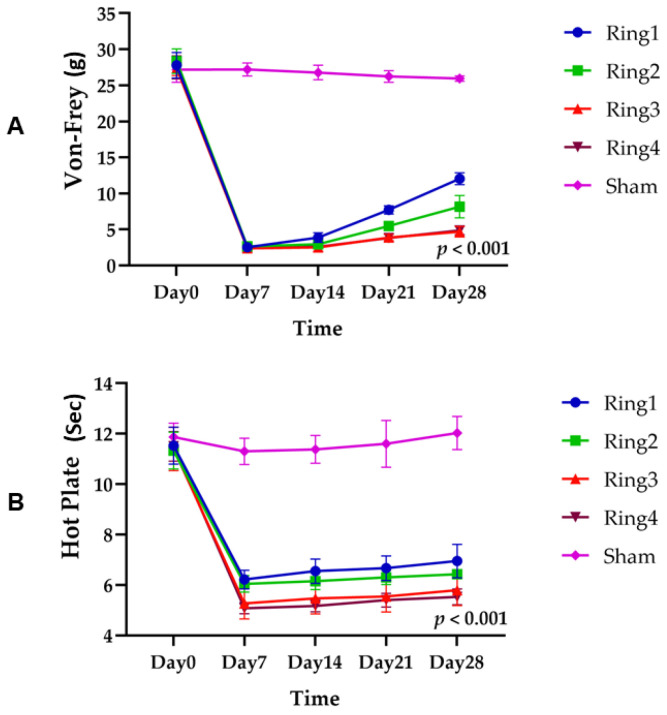
Plot of the values of thresholds in the Von Frey and hot plate tests related to different severities of nerve damage at the different time points. Sprague–Dawley rats (250–300 g) were randomly assigned to five groups: sham, and one, two, three, or four 3-0 chromic gut ligatures loosely tied around the left sciatic nerve. Von Frey and hot plate tests were measured 3 days before the ligation, and continuous measurements were made once a week for 4 weeks post-surgery until the end of the experiments. (**A**) Plot of withdrawn threshold in gm using the Von Frey test in different treatment groups related to different time points. (**B**) Plot of withdrawn threshold in seconds using the hot plate test in different treatment groups related to different time points. Sham, Ring 1, Ring 2, Ring 3, and Ring 4: see text. Statistical analysis by repeated-measures ANOVA. *n* = 6 per group.

CCI induced a sustained increase in mechanical allodynia and thermal hyperalgesia. Specifically, a progressive reduction in the withdrawal threshold for mechanical stimuli was observed with increasing numbers of ligatures, from one to four (Figure 10A). Similarly, thermal sensitivity exhibited a gradual decline in withdrawal latency, thereby indicating an intensification of hyperalgesia with escalating nerve constriction (Figure 10B). However, no significant differences were observed between the Ring 3 and Ring 4 groups at postoperative days 21 and 28 in either the Von Frey or hot plate tests. This phenomenon may be attributable to partial spontaneous functional recovery following nerve injury during the later stages of observation.

## 3. Discussion

### 3.1. Hyperspectral Imaging as an Objective Tool for Neuropathic Pain Assessment

Peripheral nerve injury often leads to skin denervation, pain hypersensitivity, and neuropathic pain. Aberrant reinnervation patterns likely sustain these symptoms. While most pain assessments rely on subjective reporting of symptoms, they are better complemented by objective clinical findings and, when appropriate, confirmatory diagnostics. Hyperspectral imaging (HSI), an emerging non-invasive modality, captures transcutaneous spatial and spectral data information across numerous wavelengths. Among these, we found that the 1100 nm wavelength demonstrated the highest reliability in predicting injury severity, whereas the 1300 nm wavelength showed significant deviations only in advanced cases. Therefore, HSI at 1100 nm may serve as a rapid, objective, and quantitative tool for evaluating neuropathic pain severity.

### 3.2. Validation of the Chronic Constriction Injury Model for Graded Neuropathic Pain

The most used models of neuropathic pain involve altered hindlimb cutaneous sensory thresholds following partial peripheral nerve injury. Among these, the chronic constriction injury (CCI) model of the sciatic nerve [26], the partial sciatic nerve ligation (PNL) model [30] and the spinal nerve ligation (SNL) model [29] are the most widely adopted. The CCI model that we used has significantly advanced neuropathic pain research, as the placement of loose chromic ligatures around the rat sciatic nerve elicits behavioral responses analogous to human neuropathic pain [26]. A previous study employing varying numbers of ligatures in the chronic constriction injury (CCI) model demonstrated that the severity of neurobehavioral alterations was strongly correlated with histomorphological changes along the neuroaxis, from the paw skin to the somatosensory cortex [28]. We acknowledge the ongoing debate regarding the extent of neurobehavioral deficits induced by different numbers of nerve constriction ligatures in rat versus mouse models [33]. Therefore, the CCI model—particularly when designed with graded levels of constriction—represents a valuable preclinical paradigm for evaluating the potential of hyperspectral imaging (HSI) to quantify the severity of neuropathic pain.

### 3.3. Spectral Information Divergence Analysis and Hyperspectral Data Processing

Non-invasive hyperspectral imaging (HSI) captures both spatial and spectral information across wavelengths, detecting subtle biochemical and physiological changes in living tissues [34]. Regarding neuropathic pain, HSI is known for its potential to visualize transcutaneous alterations associated with peripheral nerve injury. Such alterations include microvascular dysfunction, inflammation, and metabolism [35]. They often precede overt clinical manifestations, making HSI a valuable tool for early detection and monitoring of neuropathic conditions [36,37]. Clinical studies have identified seven specific wavelengths (1100, 1150, 1200, 1300, 1450, 1550, and 1650 nm) as being particularly effective for detecting diabetic neuropathy in patients with diabetes [37]. The correlation between HSI-derived spectral signatures and behavioral pain responses therefore suggests the potential of HSI as an objective and quantitative biomarker for assessing grade of neuropathic pain.

Compared with conventional tools of assessment that largely depend on subjective patient reports, HSI provides a high-resolution, operator-independent method suitable for longitudinal monitoring of neuropathic progression and therapeutic responses [34]. In this study, we created various grades of chronic constriction injury (CCI) to induce corresponding degrees of somatosensory damages along the peripheral to central pathways [28]. This model was used to evaluate the responsiveness of HSI to escalating neuropathic pain scores. Our findings revealed that the 1100 nm wavelength was the most powerful predictor of injury severity from subtle to severe, while significant deviations at the 1300 nm wavelength were observed only in more severe cases. Results further corroborate the clinical applicability of specific wavelengths, particularly those previously identified for diabetic neuropathy detection [37,38].

Hyperspectral image classification applied in the medical field uses models primarily based on either pixel-wise or subpixel-wise classification, depending on pixel information. The method of pixel-wise classification is divided into either parametric or nonparametric. Parametric classifiers typically assume that the data follow a normal distribution, an assumption that is frequently violated in real-world biomedical datasets [38]. Commonly employed supervised classification techniques include support vector machines, artificial neural networks, spectral information divergence (SID), and spectral angle mapper [39]. SID, in particular, models the spectrum of a hyperspectral image pixel according to a probability distribution, enabling the discrepancy between two spectra measured in terms of probabilistic behavior to be revealed [39]. In this study, we utilized the SID method to calculate alterations in paw skin associated with various grades of nerve injury along different time points. Our findings suggest that SID provides a reliable and robust method for hyperspectral data processing in the evaluation of neuropathic changes.

### 3.4. Histopathological and Neuroinflammatory Alterations Associated with Neuropathic Pain

Manipulation of chronic constriction nerve injury severity by increasing the number of ligature rings has resulted in progressively escalated histomorphological alterations, spanning from the paw skin to the central nervous system, and this closely parallels the degree of neurobehavioral impairment [28]. Nerve growth factor (NGF) plays a critical role in peripheral nociception, and its expression levels are highly correlated with the severity of nerve injury [26,28,40]. In contrast, reduced expression of protein gene product 9.5 (PGP 9.5) in the axons of the epidermis and dermis has been associated with neuropathic pain [26], whereas restoration of PGP 9.5-positive fibers indicates recovery from neuropathic conditions [32]. Dorsal root ganglion (DRG), located within the intervertebral foramina of the spinal cord, comprises sensory neurons that respond dynamically to peripheral nerve injury. In this context, increased expression levels of TNF-α, NGF, and synaptophysin observed in both in vitro and in vivo models reflected the grade of neuropathic pain [28,32]. Furthermore, attenuation of these inflammatory mediators was found to parallel severity reductions in neuropathic pain [28,32,41,42]. In addition, microglia in the dorsal spinal cord rapidly undergo morphological changes, proliferation, and transition to an activated state following peripheral nerve injury [43]. This activation of microglia contributes to the sensitization of dorsal horn neurons, enhancing pain transmission, and finally leading to hyperalgesia and allodynia, hallmark features of neuropathic pain [44]. Together, these well-established pathological parameters—induced progressively by escalated nerve constriction—from paw skin alteration to dorsal spinal cord changes, provided the biological basis for assessing different severities of nerve injury using HSI.

### 3.5. Denervated Muscle Degeneration and Regenerative Responses Following Nerve Injury

Chronic constriction injury (CCI) is known to not only affect sensory nerves but also motoneurons, suggesting that pathological changes in the anterior horn of the spinal cord should be considered in evaluations of nerve injury [45]. The severity of nerve damage is closely correlated with the extent of injury in denervated muscle. Muscle functional recovery following nerve injury involves neurotrophic factors that facilitate axonal transport, such as brain-derived neurotrophic factor (BDNF) [46]. Growth-associated protein 43 (GAP-43), which is expressed in skeletal muscle fibers, plays a pivotal role in muscle regeneration, with increased expression during the repair process [47]. Desmin, a key intermediate filament protein crucial for maintaining muscle structural integrity, has also been implicated in muscle recovery and functional restoration [47]. Additionally, acetylcholine receptor activation by motoneurons is sufficient to prevent muscle denervation [46]. Pax3-positive myosatellite progenitor cells are also restored during the regeneration of denervated muscle [46]. In the current study, denervated muscles showed significantly lowered expression of GAP-43, acetylcholine receptors, BDNF, Pax3, and Desmin, all associated with various degrees of nerve injury. Our findings further support that these different levels of nerve constriction result in distinct patterns of muscular damage, providing a robust foundation for the application of HSI in assessing neuropathic pain.

### 3.6. Potential Role of the HGF/c-Met Signaling Pathway in Nerve Injury and Repair

HGF cooperates with NGF in promoting the survival and growth of parasympathetic and proprioceptive neurons and this also occurs within the same neurons [48], and TNF-α is positive regulator of HGF expression, which contributes to HGF’s role as a regenerator of damaged tissues after the occurrence of inflammatory diseases [49]. A notable methodological limitation of the present study is the lack of analysis of the hepatocyte growth factor (HGF) signaling pathway in both injured paw skin and dorsal spinal cord tissues, despite the study aiming to correlate hyperspectral imaging (HSI) findings with molecular alterations following chronic constriction nerve injury. The HGF pathway consists of the HGF activator (proHGFA), its substrate proHGF, and the membrane receptor c-Met [50]. Upon HGF binding, c-Met undergoes autophosphorylation and activates downstream signaling cascades, including ERK/MAPK and Akt pathways, thereby regulating diverse biological processes such as angiogenesis, tissue repair, anti-apoptotic signaling, and inflammatory modulation [51]. Accumulating evidence further suggests that HGF suppresses NF-κB-mediated inflammatory responses and is positively regulated by pro-inflammatory cytokines, including IL-1α, IL-1β, TNF-α, and IFN-γ, highlighting its critical role in tissue regeneration following inflammatory injury [52,53]. In addition to its regenerative properties, HGF has been recognized as a potent neurotrophic factor capable of promoting neuronal survival, neurogenesis, neural stem cell proliferation and differentiation, dendritic remodeling, and synaptic plasticity [54,55]. Previous studies have demonstrated marked upregulation of HGF and c-Met expression following cerebral and spinal cord injury, whereas exogenous HGF administration significantly enhanced neuronal and oligodendrocyte survival, angiogenesis, and axonal regeneration while reducing glial scar formation and inflammatory cell infiltration [56,57,58]. Moreover, HGF therapy has been reported to exert anti-inflammatory, anti-fibrotic, anti-apoptotic, and anti-nociceptive effects in experimental models of peripheral nerve and spinal cord injury, including attenuation of glial activation, mechanical allodynia, and thermal hyperalgesia [59,60]. Therefore, incorporation of HGF-related mRNA and protein analyses would have substantially strengthened the mechanistic interpretation of the present HSI findings and provided additional insight into the molecular pathways underlying nerve injury and repair.

### 3.7. Limitations and Translational Considerations of the Present Study

The chronic constriction injury (CCI) model, with graded levels of nerve compression, induces graded levels of neurobehavioral deficits and histomorphological alterations, extending from the paw skin to the somatosensory cortex. This feature positions CCI as a favorable approach for evaluating novel devices aimed at assessing neuropathic pain [28]. While the model effectively replicates clinical symptoms such as allodynia and hyperalgesia, several limitations hinder its translational applicability to human neuropathic pain conditions. First, the extent of nerve injury in CCI is inherently variable due to inconsistent ligature tensions applied during surgery. This variability contributes to inconsistent outcomes in both behavioral and molecular studies. Second, although the model accurately reflects peripheral nerve damage, it fails to reproduce the complex processes of central sensitization that could play a crucial role in many central neuropathic pain syndromes. As a result, it does not capture the full pathophysiological spectrum of centrally mediated pain. Third, we found that the inflammatory response induced by CCI is largely restricted to the peripheral injury site. This localized response does not adequately represent the systemic immune–neural interactions involved in broader conditions such as diabetic neuropathy or multiple sclerosis. Fourth, behavioral assessments in the CCI model typically focus on evoked responses—such as mechanical allodynia and thermal hyperalgesia—thereby underestimating spontaneous or ongoing pain, which is a hallmark of chronic neuropathic pain in humans. Fifth, the utility of the CCI model in long-term studies is limited by the spontaneous recovery of nerve function, which may result in a gradual decline in pain-related behaviors over time. Finally, there remains no universally established biomarker for assessing the severity of neuropathic pain based on the expression of PGP 9.5 and NGF in the paw skin. In addition, the use of NGF, TNF-α, and synaptophysin expression as indicators for grading neuropathic severity remains controversial. Similarly, alterations in GAP-43, AChR α1, and Pax3 expression cannot solely serve as definitive predictors of denervated muscle injury and regeneration. Despite these limitations, the CCI model remains unique in its ability to generate graded levels of nerve injury. This characteristic provides a valuable experimental platform for evaluating the sensitivity and efficacy of novel diagnostic and therapeutic approaches in detecting and monitoring neurobehavioral alterations associated with neuropathic pain.

Although HSI does not measure neural activity directly, it can detect secondary physiological alterations in the paw skin resulting from nerve injury—such as microcirculatory dysfunction, dermal fibrosis, and neuroinflammatory changes—that correlate with the severity of neuropathic pain. These surface-level spectral changes, particularly in the 900–1700 nm SWIR range, are sensitive to variations in water and lipid absorption peaks, reflecting altered tissue hydration and metabolic status following peripheral nerve damage. HSI serves as a non-invasive optical surrogate marker for microenvironmental alterations secondary to neuropathic pain, rather than a direct detector of neural activity.

## 4. Materials and Methods

### 4.1. Animal Model for Chronic Constrictive Injury

Sprague–Dawley rats (from National Laboratory Animal Center, Taipei, Taiwan) weighing 250 to 300 g were used after receiving approval from the Institutional Animal Care and Use Committee or the Panel of Taichung Veterans General Hospital (La-1121973), in accordance with ARRIVE guidelines. Rats were first anesthetized with 4% isoflurane for induction followed by a maintenance dose (1 to 2%). Chronic constriction injury was placed around the left sciatic nerve as described earlier [28,61]. One to four loose ligatures of the 3-0 chromic gut were placed around the sciatic nerve at 1 mm intervals to produce graded degrees of constriction. The incision was subsequently closed with absorbable sutures in layers, and animals were then allowed to recover from surgery. Animals were divided into a sham group, and 4 experimental groups: Ring1, Ring 2, Ring 3, and Ring 4 (*n* = 6 for each group). For a design including five groups and repeated assessments at five time points (days 0, 7, 14, 21, and 28), the sample size was planned using a significance level of 0.05 and a desired statistical power of 80%. Based on conventional assumptions for repeated-measures designs and expected moderate-to-large biological effects, approximately 6–12 animals per group would be required. In practice, 6 animals per group is commonly used in similar exploratory animal studies.

Following surgery, recovered rats were allowed free access to food and water and were housed (2 rats/cage) in a temperature-controlled environment (20 °C) under a 12 h light/12 h dark cycle. Behavioral evaluations were performed by a technical assistant blinded to the treatment allocation. The behavioral tests were blindly conducted in the following sequence: assessment of mechanical allodynia, evaluation of thermal hyperalgesia, and analysis using the CatWalk gait system. All behavioral measurements were performed three days before nerve ligation to establish baseline values and were subsequently repeated once weekly for four consecutive weeks after surgery until the conclusion of the experiment. At designated endpoints (28 post-injury), animals were deeply anesthetized with 4% isoflurane and subsequently subjected to transcardial perfusion with 4% paraformaldehyde in 0.1 M phosphate buffer (pH 7.4) for tissue fixation, with tissue from the paw skin, dorsal root ganglion, and spinal cord collected for immunohistochemical and histological analyses.

For the survival study, animals were closely monitored at least twice daily to ensure welfare and to detect any signs of distress. Clinical observations included body weight, locomotor activity, grooming behavior, and indicators of pain or systemic illness. Predefined humane endpoints were established in accordance with animal care guidelines to minimize suffering. Animals were euthanized if any of the following criteria were met: (i) body weight loss exceeding 20% of baseline, (ii) severe motor impairment or inability to access food and water, (iii) persistent self-injury or refractory pain, (iv) signs of systemic compromise such as dyspnea or hypothermia, or (v) moribund condition as determined by a veterinarian.

### 4.2. Hyperspectral Imaging (HSI) System

An SWIR-IGA-I push broom HSI system (Isuzu Optics Corp., Hsinchu, Taiwan) was used for skin assessments. This experimental platform was used to obtain hyperspectral images of paw skins on both sides of rats with different severities of sciatic nerve injury. The imaging platform was equipped with four 50 W halogen lamps (DecoStar 51 ALU 41871 WFL 50 W, Osram, Germany) symmetrically positioned approximately 30 cm above the sample and covered with diffusion cloth to ensure uniform illumination while preventing direct light exposure to the rat (see Supplementary Figure S1). The imaging field measured approximately 24 × 25 cm^2^, and each hyperspectral acquisition was completed within 30 s. Following each imaging session, the animal was promptly removed from the platform. The surface temperature of the rat was measured before and after imaging using a non-contact infrared thermometer (FT-F41, Fudakang Industrial Co., Ltd., New Taipei City, Taiwan). The temperature difference was less than 0.7 °C, confirming that the illumination produced no measurable heating or tissue damage. The hyperspectral imaging system captured 512 spectral-band images across wavelengths ranging from 900 to 1700 nm in the near-infrared spectrum. The resulting data were used to generate average spectral profiles representing the spectra of all 512 bands from six randomly selected locations within each hyperspectral image cube. Each spectral-band image had a spatial resolution of 640 × 512 pixels. A technical assistant blinded to the treatment allocation performed the hyperspectral imaging (HSI) measurements. We aimed to measure the difference between two reflectance spectra of the paw skin across groups of rats with 4 different grades of chronic constriction injury in addition to the sham based on spectral information divergence (SID). SID is typically used to quantify similarity between sets of spectral information [37]. To measure the distance between the probability distributions generated by the spectral signatures of two-pixel vectors, $s_{i}$ and $s_{j}$, SID was calculated according to the following formula (1):

(1) $$SID\left(s_{i},s_{j}\right)=D\left(s_{i}∥s_{j}\right)+D\left(s_{j}∥s_{i}\right)$$

where $D\left(s_{i}∥s_{j}\right)$ and $D\left(s_{j}∥s_{i}\right)$ represent the average difference in $s_{j}$ relative to $s_{i}$ and $s_{i}$ relative to $s_{j}$, respectively.

The average waveform of HSI from 6 shams was used as a reference for comparison. We further analyzed the SID in paw skin across different grades of chronic constriction injury by comparing it with similar spectral information derived from sham groups.

### 4.3. Dynamic Plantar Aesthesiometer and Thermal Hyperalgesia

Mechanical withdrawal thresholds of the hind paws were assessed using an automated dynamic plantar aesthesiometer (dynamic plantar aesthesiometer; Ugo Basile Cat. No. 37450). Prior to testing, rats were acclimated for 15 min in individual transparent chambers positioned on an elevated metal mesh platform. Mechanical stimulation was applied to the plantar surface of the hind paw through the mesh floor using a 0.5 mm diameter steel filament. The device delivered a gradually increasing force from 0 to 50 g over 20 s at a constant rate of 2.5 g/s. Upon paw withdrawal, the instrument automatically terminated the stimulus, and the corresponding force was recorded with a precision of 0.1 g. Each animal underwent three consecutive measurements with intertrial intervals of 3–5 min to minimize sensitization or stress. The mean value of the three trials was calculated and used for subsequent statistical analysis [62]. Thermal nociceptive sensitivity was evaluated using the hot plate assay (Technical & Scientific Equipment GmbH, TSE systems) (Huntley, IL, USA). Animals were placed on a temperature-controlled surface maintained at 52 °C, and the paw withdrawal latency was defined as the interval between initial paw contact and withdrawal. To avoid thermal injury, a maximum cutoff time of 20 s was enforced. The recorded latency served as an index of thermal hyperalgesia [28].

### 4.4. CatWalk-Automated Quantitative Gait Analysis

The CatWalk XT system is equipped with a high-speed digital camera sampling 100 frames/s. The video camera transforms the captured scene into a digital image, and data are transferred, through an Ethernet, to a computer. The Illuminated FootprintTM enables intensity differences to be detected between animals’ paws. The 3D footprint intensity tab plots, in a 3D chart, the print intensity of the 4 paws for each individual frame in which individual paws contact the glass plate. The pixel intensity varies from 0 to 225 and is color-coded for display. A 3D chart can also be rotated for optimal viewing [63]. Quantitative analyses of the recorded data from the Catwalk XT included the following parameters: step sequence distribution, regularity (RI), print area, base of support, duration of the swing and stance phases, and hind paw pressure. All the parameters are scaled in arbitrary units (a.u.).

Step Sequence Distribution: A total of 6 distinct walking patterns, representing normal step sequences in rats, were identified, and categorized into 3 classes according to the order of paw placement.

Regularity Index (RI): Quantification of interlimb coordination was performed and was considered optimal when locomotion consisted exclusively of regular step sequences. RI was calculated using the formula RI = (NSSP × 4/PP) × 100%, where NSSP denotes the number of normal step sequence patterns and PP represents the total number of paw placements. The presence of irregular steps or additional paw placements gave a lower RI value.

Print Area: Representing the total contact area, expressed in pixels, of each paw during the stance phase. An enlarged hind paw print area likely reflects impaired plantar support or paw dragging, whereas a reduction in print area is commonly associated with mechanical allodynia.

Base of Support: Indicates the distance (in millimeters) between the two hind paws, and is calculated perpendicular to the direction of movement.

Swing and Stance Phase Durations: Parameters influenced by walking speed and neuromuscular function, and they were expressed as percentages of the total step cycle. Phase duration was calculated as (time spent in swing or stance/duration of a single step) × 100%, with absolute values recorded in second.

Hind Paw Pressure: Defined as the mean signal intensity within the contact area at the point of maximal paw–surface interaction, and it was expressed in arbitrary units (a.u.)

### 4.5. Immunohistochemistry

Animals were deeply anesthetized and transcardially perfused with physiological saline containing 0.5% sodium nitrate and heparin (10 U/mL), followed by fixation with 4% paraformaldehyde prepared in 0.1 M phosphate buffer. Tissues including plantar skin, sciatic nerve, dorsal root ganglia, gastrocnemius muscle, and the entire spinal cord (L4-6 region) (with emphasis on the posterior column and anterior horn) were harvested. Specimens were cryosectioned into 8 μm thick serial sections and mounted on Superfrost Plus slides for immunohistochemical analysis (MenzelGlaser, Braunschweig, Germany). Sections were incubated with primary antibodies targeting PGP 9.5 (Cat #ab8189, 1:100, abcam, Cambridge, UK), NGF (Cat# ab52987, 1:500, abcam, Cambridge, UK), NF(Cat# 2836, 1:500, Cell Signaling, Danvers, MA, USA), TNF-α (Cat# ab66579, 1:500, abcam, Cambridge, UK), Iba-1 (Cat# NB100-1028, 1:500, NOVUS, Centennial, CO, USA), synaptophysin (Cat# ab14692, 1:500, abcam, Cambridge, UK), Nicotinic Acetylcholine Receptor alpha 1 (AChR) (Cat# ab308306, 1:500, abcam, Cambridge, UK), Neu-N (Cat# ab177487, 1:500, abcam, Cambridge, UK), growth-associated protein 43 (GAP 43) (Cat# GTX127937, 1:250, GeneTex, Irvine, CA, USA), Desmin(Cat# ab32362, 1:500, abcam, Cambridge, UK), paired box 3 (Pax 3) (Cat# 251468, 1:100, ABBIOTEC, San Diego, CA, USA), and brain-derived neurotrophic factor(BDNF) (Cat# AB1779, 1:250, Millipore, Burlington, MA, USA). The immunoreactive signals were observed by goat anti-mouse IgG (FITC) (Jackson, 1:200 dilution) and anti-mouse IgG (Rhodamine) (Jackson, 1:200 dilution, West Grove, PA, USA). Six tissue samples from each group were sectioned into 8 μm thick slices, and six consecutive sections from each sample were subjected to immunohistochemical staining for subsequent analysis. Fluorescent images were obtained with an Olympus BX40 Research Microscope (Olympus Corporation, Tokyo, Japan), with identical laser power and exposure time. Quantitative analysis was performed using ImageJ software (version 1.52). Images were separated into individual color channels, and the relevant channel was retained for analysis. After conversion to TIFF format, thresholding was applied to isolate positively stained regions, followed by generation of binary images. In the binary images, positively stained pixels were converted to black and background pixels to white. The total number of black pixels was calculated and normalized to the tissue area (pixel density/mm^2^) to represent immunoreactive signal density [64,65]. In this study, we minimized these sources of variability by maintaining strict experimental consistency. All tissue samples were collected under identical conditions, and immunostaining was performed in parallel using the same antibody batches and standardized protocols. Quantification was conducted by blinded observers in predefined anatomical regions to ensure reproducibility.

### 4.6. Histology Examination

Following behavioral and electrophysiological assessments, 6 anesthetized rats from each group were transcardially perfused with 4% paraformaldehyde in 0.1 M phosphate buffer (pH 7.4). Their paw skin and spinal cord (dorsal column and anterior horn cells) were harvested and subjected to Masson’s trichrome staining (Cat# HT15-1KT, Sigma-Aldrich, St. Louis, MO, USA) and Nissl staining (Cat # PS1010, Thionin, St. Louis, MO, USA). The severity of fibrosis and counts of Nissl staining in the dorsal spinal cord and anterior horn region were measured by the method described in the above section [64,65]. Paw skin samples were fixed in 4% paraformaldehyde, embedded in paraffin, and sectioned into 5 μm slices. Six tissue samples from each group were sectioned into 5 μm thick slices, and six consecutive sections from each sample were subjected to immunohistochemical staining for subsequent analysis. The sections were stained using Masson’s trichrome staining according to the manufacturer’s protocol to evaluate collagen deposition and dermal fibrosis. In this staining method, collagen fibers were stained blue, muscle and cytoplasm were stained red, and nuclei were stained dark purple/black. Stained sections were examined under a light microscope, and representative images were captured under identical magnification and exposure settings for comparison among experimental groups. Spinal cord tissues were fixed in 4% paraformaldehyde, embedded in paraffin, and sectioned into 5 μm slices. The sections were subjected to Nissl staining using cresyl violet solution to evaluate neuronal morphology and neuronal survival within the spinal cord. Briefly, the sections were deparaffinized, rehydrated, and stained with cresyl violet, followed by dehydration and cover slipping. Nissl bodies within neuronal cytoplasm were visualized under a light microscope, and representative images were captured under identical magnification and exposure settings for comparison among experimental groups. In the measurement of Massons’ trichrome staining, digital images were captured under identical exposure and illumination settings. The collagen-positive area, stained blue, was quantified using ImageJ software. The region of interest was defined as the dermal area beneath the epidermis. After color deconvolution or color threshold adjustment, the blue-stained collagen fibers were selected and measured. Quantitative analysis was performed using ImageJ software (version 1.52). Images were separated into individual color channels, and the relevant channel was retained for analysis. After conversion to TIFF format, thresholding was applied to isolate positively stained regions, followed by generation of binary images. In the binary images, positively stained pixels were converted to black and background pixels to white. The total number of black pixels was calculated and normalized to tissue area (pixel density/mm^2^) to represent immunoreactive signal density [64,66]. All image analyses were performed by a technical assistant blinded to the treatment allocation to ensure unbiased quantification.

### 4.7. Electrophysiological Assessments

Compound muscle action potentials (CMAPs) were recorded from the gastrocnemius muscle to evaluate neuromuscular function, with both peak amplitude and conduction latency analyzed. A monopolar recording needle electrode was inserted approximately 10 mm distal to the tibial tubercle, while a reference monopolar electrode was positioned about 20 mm apart. Electrical stimuli (5 mA; 20–200 Hz) were applied via a stimulating electrode placed proximal to the site of nerve injury. The amplitude and latency values obtained from the injured limb were normalized to those of the contralateral, uninjured side and expressed as ratios to minimize inter-animal variability. All electrophysiological assessments were conducted by a trained technician who was blinded to group assignment, thereby ensuring unbiased data acquisition, and enhancing the reliability and reproducibility of the measurements [64,65]. All electrophysiological measurements were performed by a technical assistant who was blinded to the treatment allocation to ensure objectivity and reproducibility.

### 4.8. Statistical Analysis

All data are expressed as the mean ± standard error of the mean (SEM). Prior to analysis, data distribution was verified for normality. Two-way repeated-measures ANOVA was applied to compare group differences in hyperspectral imaging (HSI) values, mechanical allodynia, thermal hyperalgesia, and CatWalk gait parameters. One-way ANOVA was applied for comparisons of histological and immunohistochemical signal densities, muscle weight, compound muscle action potential (CMAP) amplitude, and nerve conduction latency among groups. Post hoc multiple comparisons were performed using Bonferroni correction. Statistical analyses were conducted using SPSS software (version 25; IBM Corp., Armonk, NY, USA), and a *p* value of <0.05 was considered statistically significant.

## 5. Conclusions

Different graded levels of nerve compression induced significantly varied severities of histomorphological alterations, extending from the paw skin to the dorsal spinal cord, and they were also paralleled by distinct neurobehavioral changes. Using this model, hyperspectral imaging (HSI) of the paw skin demonstrated robust discriminatory power in detecting graded nerve injury, particularly at the 1100 nm wavelength. These findings support the potential of HSI as a non-invasive tool for the detection and evaluation of neuropathic pain.

## Figures and Tables

**Table 1:** Immunohistochemistry alteration related to different numbers of rings in paw skin.

	Sham	Ring 1	Ring 2	Ring 3	Ring 4	*p* Value
Masson’s Trichrome	8717.7 ± 368.6	9669.8 ± 1476.3	13,878.1 ± 346.4	17,258.9 ± 502.5	22,290.4 ± 836.7	<0.01
PGP 9.5	6887.8 ± 438.9	4961.5 ± 205.9	3711.1 ± 132.9	2719.3 ± 213.5	1426.1 ± 179.3	<0.01
NGF	339.7 ± 38.3	792.1 ± 85.4	1318.7 ± 55.8	2121.8 ± 91.8	3299.1 ± 404.2	<0.05

The measurement unit was presented as pixel/mm^2^. The data were presented as mean ± standard error. *n* = 6.

**Table 2:** Microglia in the dorsal horn related to different treatments.

	Sham	Ring 1	Ring 2	Ring 3	Ring 4	*p* Value
Mean	5.2	56.3	76.2	169.1	233.8	<0.01
SE	0.9	4.7	2.7	10.2	16.1	

The measurement unit was presented as counts of microglia/mm^2^; *n* = 6.

## Data Availability

The original contributions presented in this study are included in the article/Supplementary Materials. Further inquiries can be directed to the corresponding author.

## Supplementary Materials

The following supporting information can be downloaded at: https://www.mdpi.com/article/10.3390/ijms27125164/s1.

## Abbreviations

HSI	hyperspectral imaging
CCI	chronic constrictive injury
NGF	nerve growth factor
PGP 9.5	protein gene product 9.5
TNF-α	tumor necrosis factor-alpha
AChRs	acetylcholine receptors
GAP-43	growth-associated protein 43
Pax3	paired box protein 3
BDNF	brain-derived neurotrophic factor
DN4	Douleur Neuropathique 4
LANSS	Leeds Assessment of Neuropathic Symptoms and Signs
QST	quantitative sensory testing
IENFD	intraepidermal nerve fiber density
TrkA	tropomyosin receptor kinase A
CGRP	calcitonin gene-related peptide
SID	spectral information divergence
CMAP	muscle action potential
PNL	partial sciatic nerve ligation
SNL	spinal nerve ligation
DRG	dorsal root ganglion
