# Supplementary material for: Validation of Paw Skin Hyperspectral Imaging for Assessing Neuropathic Pain Severity in a Chronic Constriction Injury Model
Source: Int J Mol Sci. 2026 Jun 6;27(12):5164. doi: 10.3390/ijms27125164 (PMC13300190; doi:10.3390/ijms27125164)
Supplement: Supplementary file 1 [file ijms-27-05164-s001.zip › ijms-4251887-supplementary.pdf]

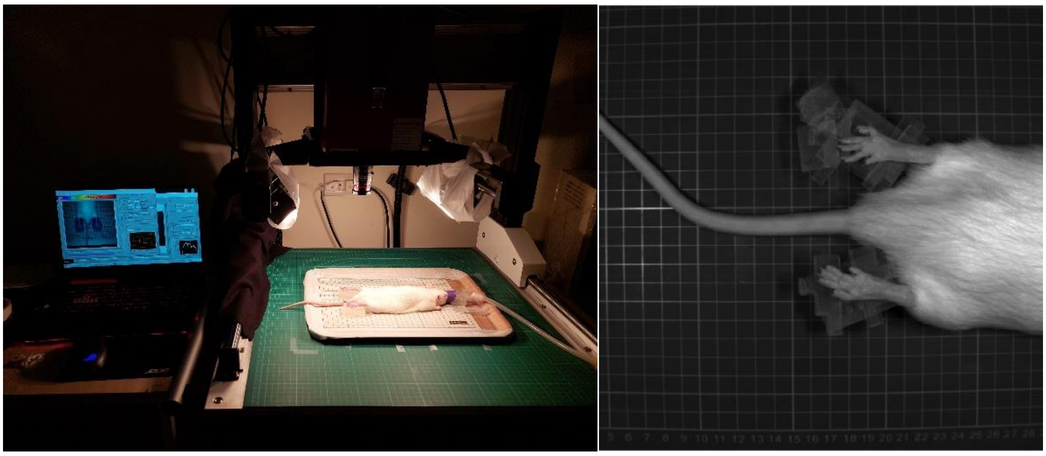

(a) (b)

Supplementary Figure 1: Setup and imaging area of the SWIR hyperspectral imaging system.

(a) Experimental platform equipped with four 50 W halogen lamps covered with diffusion cloth to ensure uniform illumination and prevent direct light exposure to the rat.

(b) Representative grayscale image from one SWIR spectral band (1092nm) showing the actual imaging area of the rat paws.
